# Outcomes of multiple gestation births compared to singleton: analysis of multicenter KID database

**DOI:** 10.1186/s40748-021-00135-5

**Published:** 2021-10-28

**Authors:** Renjithkumar Kalikkot Thekkeveedu, Nilesh Dankhara, Jagdish Desai, Angelle L. Klar, Jaimin Patel

**Affiliations:** Newborn Medicine, University of Mississippi Medical Center, 2500 N State St, W154, Jackson, MS 39216 USA

**Keywords:** Neonates, Outcomes, Twins, Triplets, Higher-order multiples

## Abstract

**Background:**

The available data regarding morbidity and mortality associated with multiple gestation births is conflicting and contradicting.

**Objective:**

To compare morbidity, mortality, and length of stay (LOS) outcomes between multiple gestation (twin, triplet and higher-order) and singleton births.

**Methods:**

Data from the national multicenter Kids’ Inpatient Database of the Healthcare Cost and Utilization Project from the years 2000, 2003, 2006, 2009, 2012, and 2016 were analyzed using a complex survey design using Statistical Analysis System (SAS) 9.4 (SAS Institute, Cary NC). Neonates with ICD9 and ICD10 codes indicating singletons, twins or triplets, and higher-order multiples were included. Mortality was compared between these groups after excluding transfer outs to avoid duplicate inclusion. To analyze LOS, we included inborn neonates and excluded transfers; who died inpatient and any neonates who appear to have been discharged less than 33 weeks PMA. The LOS was compared by gestational age groups.

**Results:**

A total of 22,853,125 neonates were analyzed for mortality after applying inclusion-exclusion criteria; 2.96% were twins, and 0.13% were triplets or more. A total of 22,690,082 neonates were analyzed for LOS. Mean GA, expressed as mean (SD), for singleton, twins and triplets, were 38.30 (2.21), 36.39 (4.21), and 32.72 (4.14), respectively. The adjusted odds for mortality were similar for twin births compared to singleton (aOR: 1.004, 95% CI:0.960–1.051, *p* = 0.8521). The adjusted odds of mortality for triplet or higher-order gestation births were higher (aOR: 1.33, 95% CI: 1.128–1.575, *p* = 0.0008) when compared to the singleton births. Median LOS (days) was significantly longer in multiple gestation compared to singleton births overall (singletons: 1.59 [1.13, 2.19] vs. twins 3.29 [2.17, 9.59] vs. triplets or higher-order multiples 19.15 [8.80, 36.38], *p* < .0001), and this difference remained significant within each GA category.

**Conclusion:**

Multiple gestation births have higher mortality and longer LOS when compared to singleton births. This population data from multiple centers across the country could be useful in counseling parents when caring for multiple gestation pregnancies.

**Supplementary Information:**

The online version contains supplementary material available at 10.1186/s40748-021-00135-5.

## Introduction

The United States and other developed countries have witnessed an increased prevalence of multiple gestation births in the late twentieth century and the first decade of the twenty-first century [1–5], which is primarily attributed to the evolution of assisted reproductive techniques [6–8]. Data from many developed countries have shown that while there is a significant drop in the number of triplet or higher-order multiple births, the number of twin births has remained stable or continued to rise [9–13].

Multiple gestation births account for 3–4.5% of all births [11, 14], are more likely to be associated with premature births [15, 16], and more commonly result in babies who are small for gestational age (SGA) and also low birth weight (LBW) [17–20]. Such factors may play a role in the outcomes of multiple gestation births, but little information is available in published literature. The published data regarding morbidity and mortality associated with multiple gestation births is conflicting and contradicting [6, 15, 18, 21–23]. .For example, Shinwell [21] and Martin et al. [18] have reported a higher incidence of mortality among twins and triplets, whereas shah et al. [24] and Garite et al. [20] found no such difference.

Most of the previous studies include a select few centers that are primarily academic and are also limited by relatively small sample sizes [23, 25], therefore, results may not be generalizable. There are two multicountry collaboration studies, neither of which include outcomes of neonates born in the United States [24, 25]. There is a need for data from a representative population-based sample in the United States to address the impact of multiple gestation births on outcomes while controlling for various baseline characteristics.

The objective of this study is to compare morbidity, mortality, and LOS outcomes between multiple gestation and singleton births among neonatal discharges in a nationally representative large dataset.

## Materials/subjects and methods

### Study design and participants

We performed a retrospective study using the Kid’s Inpatient Database (KID) [26]. The KID is a population-based administrative database compiled by the Healthcare Cost and Utilization Project (HCUP) of the Agency for Healthcare Research and Quality (AHRQ), which includes the largest collection of longitudinal hospital care data in the United States. We obtained and analyzed discharge records for the years 2000, 2003, 2006, 2009, 2012, and 2016.

Our cohort extraction started with identifying neonatal age admissions through the first 28 days after birth among all discharge records. Discharge records were excluded if (i) there was no ICD code specifying singleton versus multiple gestation births, (ii) there was the presence of congenital anomalies described in Additional file 1: Appendix 1 were present, or (iii) there were indicators for “transfer out” in order to avoid counting the same patient twice. Theresulting cohort was analyzed for morbidity and mortality as well as the odds ratio of being born at a premature GA.

**Fig. 1:**
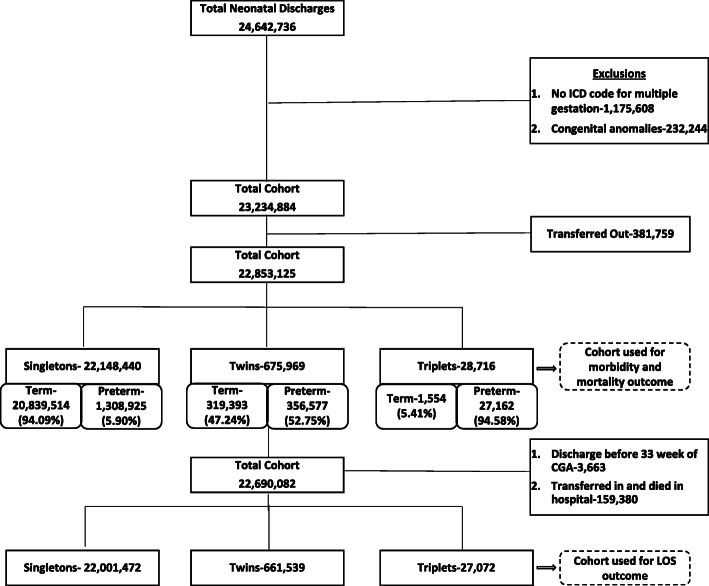
Cohort extraction

For the LOS analysis, discharge records with indicators for both “transfer in” or “transfer out” were excluded since they may result in inaccurate estimates of the LOS. The patients who appeared to have been discharged prior to 33 weeks Post Menstrual Age (PMA) were also excluded. Since premature infants do not achieve feeding competency in terms of a coordinated suck and swallow prior to 33 weeks PMA, discharge records for such patients likely represent erroneous data and may also result in inaccurate estimates of the LOS. The number of discharge records at each stage of cohort extraction is presented in Fig. 1.

Once the cohort was identified, baseline characteristics (delivery type, GA, intrauterine growth restriction (IUGR) or SGA status, sex, race, payer) and hospital characteristics (location of hospital, hospital region and hospital bedsize) were extracted for all three groups (singleton, twin, and triplet or higher-order births). Common neonatal morbidities such as respiratory distress syndrome (RDS), pulmonary hemorrhage, pneumothorax, bacterial sepsis, necrotizing enterocolitis (NEC), intraventricular hemorrhage (IVH), periventricular leukomalacia (PVL), retinopathy of prematurity (ROP), and bronchopulmonary dysplasia (BPD) were compared. A list of ICD-9-CM and ICD-10-CM codes for singleton, twin, and triplet or higher-order multiple births, all inclusion-exclusion criteria, baseline characteristics, morbidities, and outcome measures can be found in Additional file 1: Appendix 1. Our study was determined to be exempt by the University of Mississippi Medical Center Institutional Review Board.

### Statistical analysis

Data use agreement training was completed by each author who analyzed the data. Data were analyzed using SAS 9.4 while accounting for a complex survey sample design of the HCUP dataset. We utilized weighted, stratified analysis using SAS survey procedures and reported ‘n’ as weighted cases in each group.

Univariate analysis was performed for descriptive statistics and to compare baseline characteristics between the three groups, and *p*-values for chi-square tests were obtained. The whole cohort was divided into preterm (< 37 weeks) and term (≥37 weeks). Most GA ICD-9-CM codes span 2 weeks. The presumed mean GA was taken for each ICD-9-CM code to calculate the overall GA mean for the three groups. For term births, taking CDC data into consideration, a mean GA of 38.5 was applied [27]. The exact values for each GA category are available in Additional file 1: Appendix 2. Rates and odds of having preterm birth were derived in each group. The preterm morbidity outcomes were compared by logistic regression analysis while controlling for baseline characteristics and hospital characteristics. Each analysis utilized the 2-tailed statistical test with an alpha level of 0.05. Due to known multicollinearity between GA and birth weight (BW), only GA was used in the models along with IUGR or SGA status.

Mortality was compared separately in the preterm and term cohorts. For mortality, we obtained an adjusted odds ratio (aOR) for multiple gestation births compared to singleton using survey logistic regression. In preterm infants, mortality odds were adjusted for baseline demographic characteristics and comorbidities. Early neonatal deaths were defined in our study as neonatal deaths occurring in the first week of life, consistent with the World health organization (WHO) definition [28]. Since a large number of neonatal deaths in each GA category occur as early neonatal deaths, we compared these early deaths among the three groups separately in addition to comparing their overall in-hospital mortality. We excluded patients marked “transfer in” for early mortality analysis as the day of life for death is not available in the dataset.

For the LOS analysis, we compared medians (IQR) among the three groups. The LOS of each GA group was also compared, accounting for complex survey design using clusters, stratum and weights.

## Results

### Cohort extraction

Baseline characteristics of the cohort are presented in Table 1.

**Table 1:** Cohort extraction: Baseline characteristics of the cohort

Characteristic	Category	Singletons	Twins	Triplets or More	***p***-value
Gestational age	< 24 weeks	20,277 (0.09%)	4816 (0.71%)	713 (2.48%)	
	24 weeks	7458 (0.03%)	1873 (0.28%)	245 (0.85%)	<.0001
	25–26 weeks	18,338 (0.08%)	4694 (0.69%)	676 (2.35%)	
	27–28 weeks	26,293 (0.12%)	7876 (1.17%)	1325 (4.62%)	
	29–30 weeks	40,542 (0.18%)	13,722 (2.03%)	2526 (8.80%)	
	31–32 weeks	81,279 (0.37%)	31,474 (4.66%)	4741 (16.51%)	
	33–34 weeks	224,024 (1.01%)	77,985 (11.54%)	6888 (23.99%)	
	35–36 weeks	603,530 (2.72%)	149,048 (22.05%)	3735 (13.01%)	
	Preterm but GA Unknown	287,185 (1.30%)	65,088 (9.63%)	6312 (21.98%)	
	> 37 weeks	20,839,514 (94.09%)	319,393 (47.25%)	1554 (5.41%)	
	Total	22,148,440 (100.00%)	675,969 (100.00%)	28,716 (100.00%)	
IUGR or SGA	Yes	367,274 (1.66%)	45,653 (6.75%)	1856 (6.46%)	<.0001
Sex	Female	10,843,182 (49.02%)	336,883 (49.89%)	14,295 (49.83%)	<.0001
	Male	11,274,557 (50.98%)	338,380 (50.11%)	14,393 (50.17%)	
	Total	22,117,739 (100.00%)	675,263 (100.00%)	28,687 (100.00%)	
Race/Ethnicity	White	9,579,294 (52.79%)	330,163 (59.27%)	16,247 (70.15%)	<.0001
	Black	2,461,724 (13.57%)	85,412 (15.33%)	2306 (9.96%)	
	Hispanic	4,004,551 (22.07%)	82,669 (14.84%)	2435 (10.51%)	
	Asian/Pacific Islander	881,691 (4.86%)	23,992 (4.31%)	833 (3.60%)	
	Native American	124,300 (0.69%)	3261 (0.59%)	105 (0.45%)	
	Other	1,094,195 (6.03%)	31,571 (5.67%)	1236 (5.34%)	
	Total	18,145,755 (100.00%)	557,068 (100.00%)	23,161 (100.00%)	
Primary expected payer	Medicare	47,207 (0.21%)	1380 (0.20%)	21 (0.07%)	<.0001
	Medicaid	9,344,270 (42.28%)	223,397 (33.11%)	5174 (18.05%)	
	Private insurance	11,057,438 (50.03%)	409,725 (60.72%)	22,171 (77.36%)	
	Self-pay	1,030,075 (4.66%)	20,115 (2.98%)	392 (1.37%)	
	No charge	38,623 (0.17%)	683 (0.10%)	29 (0.10%)	
	Other	582,632 (2.64%)	19,455 (2.88%)	874 (3.05%)	
	Total	22,100,246 (100.00%)	674,755 (100.00%)	28,661 (100.00%)	
Region of hospital	Northeast	3,672,279 (16.58%)	133,246 (19.71%)	6370 (22.18%)	<.0001
	Midwest	4,767,208 (21.52%)	151,710 (22.44%)	7074 (24.63%)	
	South	8,412,784 (37.98%)	243,999 (36.10%)	9614 (33.48%)	
	West	5,296,168 (23.91%)	147,014 (21.75%)	5657 (19.70%)	
	Total	22,148,440 (100.00%)	675,969 (100.00%)	28,716 (100.00%)	
Hospital type	Rural	2,697,153 (12.28%)	50,055 (7.48%)	467 (1.66%)	<.0001
	Urban nonteaching	8,756,542 (39.88%)	231,462 (34.61%)	6694 (23.80%)	
	Urban teaching	10,502,357 (47.83%)	387,322 (57.91%)	20,962 (74.54%)	
	Total	21,956,052 (100.00%)	668,839 (100.00%)	28,123 (100.00%)	
Bed size of hospital	Small	2,524,672 (11.50%)	59,158 (8.84%)	1227 (4.36%)	<.0001
	Medium	5,918,612 (26.96%)	165,112 (24.69%)	5131 (18.24%)	
	Large	13,512,768 (61.54%)	444,569 (66.47%)	21,765 (77.39%)	
	Total	21,956,052 (100.00%)	668,839 (100.00%)	28,123 (100.00%)	
Delivery type	NSVD	15,728,662 (71.01%)	192,045 (28.41%)	1686 (5.87%)	<.0001
	CS	6,419,778 (28.99%)	483,924 (71.59%)	27,030 (94.13%)	
	Total	22,148,440 (100.00%)	675,969 (100.00%)	28,716 (100.00%)	

#### Prematurity

The preterm birth rates for singleton, twin, and triplet births were 6.7, 54.9 and 94.5%, respectively. Odds of being born preterm for twin births when compared to singleton was OR: 17.79 (95% CI: 17.513–18.085, *p* < 0.001). Mean GA, expressed as mean (SD), for singleton, twin and triplet births were 38.30(2.21), 36.39 (4.21), and 32.72(4.14), respectively (Table 1).

#### Morbidities

Neonatal morbidities for preterm birthswere compared among the three groups (Table 2). Interestingly, the odds of having RDS was significantly lower in twin (0.74) and triplet (0.89) preterm births when compared to singleton, while the odds of pulmonary hemorrhage was higher in twin (1.128) and triplet(1.449) preterm births when compared to singleton. The odds of BPD, any IVH, and bacterial sepsis were lower in twin births when compared to singleton, while the odds of severe IVH were higher in twin births when compared to singleton (compared to singletons, the odds of any IVH were 0.92 among twins and 0.95 among triplets). Triplet and higher-order multiple births had a greater odds of pulmonary hemorrhage, BPD, and any ROP when compared to singleton births. There was no difference in severe ROP and NEC between the three groups.

**Table 2:**
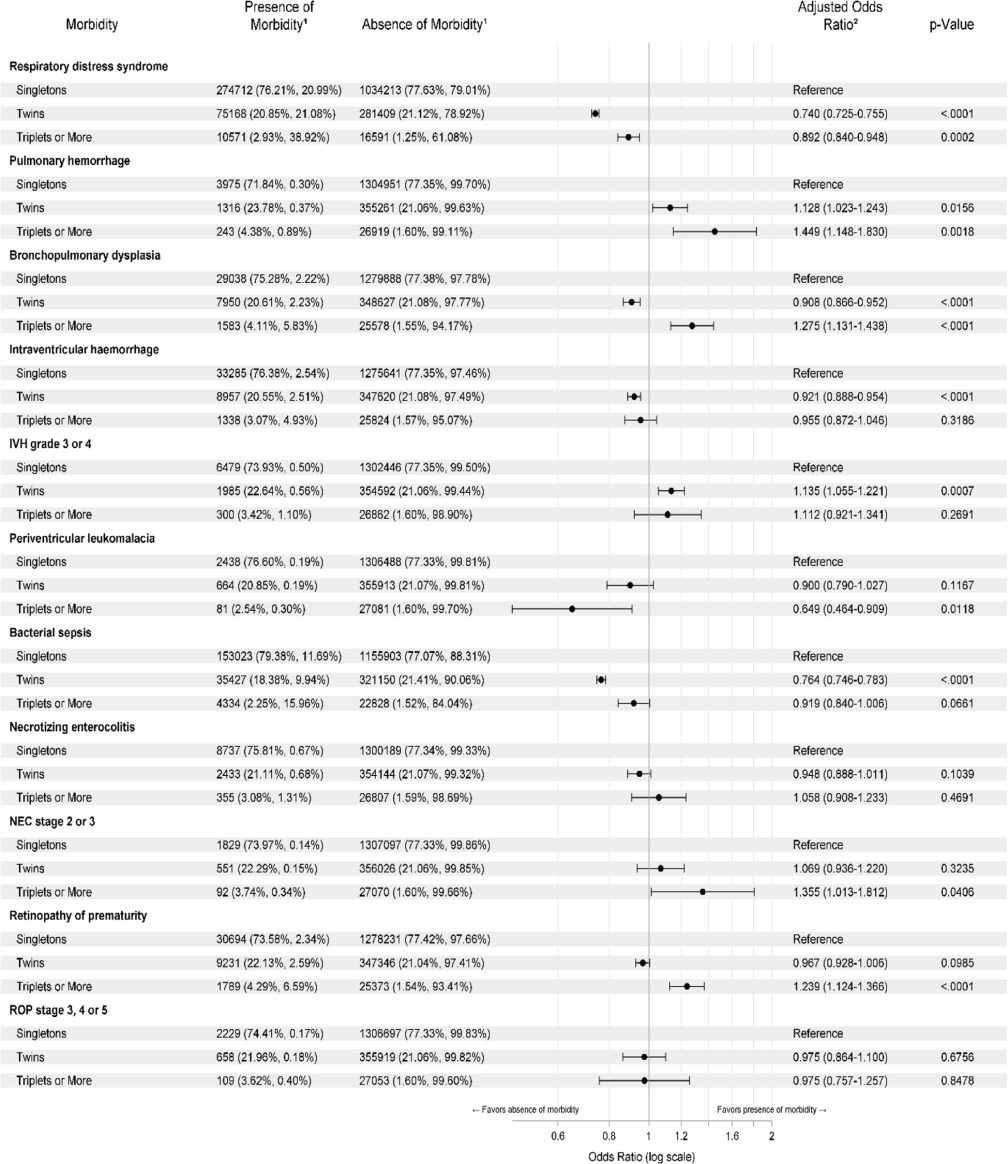
The results of comparison of the rate of morbidities between three groups after controlling for baseline characteristics in multivariate survey logistic models with adjusted odds ratios (referenced as a singleton) in the forest plot along with significant *p*-values

^1^Weighted frequency (column percent, row percent)

^2^Odds ratio adjusted for baseline characteristics including gestational age, IUGR or SGA, gender, race, primary expected payer, the region of hospital, hospital type, bedsize of the hospital and delivery type

#### Mortality

Mortality rates among preterm infants were 3.01, 2.65, and 5.07% for singleton, twins, and triplets and higher-order multiple gestation births respectively. The aORfor mortality were similar for twin births compared to singleton (1.004, 95% CI:0.960–1.051, *p* = 0.8521). The aOR for mortality for triplet or higher-order multiple gestation births was higher (aOR: 1.33, 95% CI: 1.128–1.575, *p* = 0.0008) when compared to singleton. Early mortality rates were 2.48, 2.18, and 4.15% in singleton, twin, and triplet or higher-order multiple gestation births, respectively. The aOR for early mortality were similar for twin births (1.02, 95% CI:0.967–1.074, *p* = 0.4798) and higher for triplet or higher-order multiple gestation births (1.424, 95% CI: 1.169–1.735, *p* = 0.0005) whencompared to singleton births.

The mortality rates among term infant births were 0.03, 0.24, and 5.24%, respectively, for singletons, twin, and triplet or higher-order multiple gestation births. The aOR for mortality in term twin neonates was higher (5.263, 95% CI: 4.524–6.122, *p* < 0.0001) when compared to singletons. Since most triplets are born premature, we did not compare the mortality in triplet against singletons.

#### Length of stay

Median LOS was significantly longer in all multiple gestation births when compared to singletonoverall (Median Days [IQR]: Singletons: 1.59 [1.13, 2.19] vs. Twins 3.29 [2.17, 9.59] vs. Triplets or higher 19.15 [8.80, 36.38], *p* < .0001), and this difference remained significant within each GA category (Table 3).

**Table 3:** The results of comparison of median length of stay for singletons, twins, and higher order gestations after controlling for baseline characteristics

Gestational age	Singleton Median [IQR]	Twin Median [IQR]	Triplet or More Median [IQR]	***p***-Value
< 24 weeks	118.25 [102.89, 135.90]	121.74 [108.98, 144.31]	119.13 [103.12, 133.52]	0.0314
24 weeks	105.64 [92.39, 122.88]	112.53 [97.01, 130.71]	118.84 [99.34, 139.54]	<.0001
25–26 weeks	86.80 [73.97, 102.63]	89.66 [76.71, 106.02]	93.50 [81.58, 112.50]	<.0001
27–28 weeks	63.58 [53.24, 76.24]	65.44 [54.93, 77.99]	67.18 [57.36, 81.36]	<.0001
29–30 weeks	43.13 [35.17, 52.99]	44.68 [36.69, 54.57]	45.90 [38.58, 55.91]	<.0001
31–32 weeks	24.94 [18.46, 33.10]	26.93 [20.60, 34.64]	28.91 [22.62, 36.25]	<.0001
33–34 weeks	11.11 [6.62, 16.65]	13.32 [8.89, 18.52]	15.45 [11.26, 20.73]	<.0001
35–36 weeks	2.53 [1.59, 5.06]	3.39 [2.35, 6.46]	5.02 [3.39, 9.99]	<.0001
> 37 weeks	1.57 [1.12, 2.05]	2.53 [1.80, 3.31]	3.51 [2.82, 5.51]	<.0001
Preterm GA Unknown	5.81 [2.50, 13.67]	10.39 [3.45, 33.46]	38.63 [18.92, 69.64]	<.0001

## Discussion

In this study, we compared birth GA, morbidities, mortality, and LOS between singletonand multiple gestation births among neonatal discharges from 2000 through 2016 utilizing the KID, which is a large population-based administrative database compiled by the HCUP of AHRQ. This report is the largest one of its kind, representing > 4000 hospitals and > 22 million discharge records over the last two decades. Our analysis controlled for baseline characteristics and previously identified variables that could affect the outcome, such as IUGR or SGA status. We believe these results are widely generalizable and could be of benefit with perinatal counseling in the case of multiple gestation pregnancies.

In our study, the percentages of premature babies in each GA category from 24 to 34 weeks were significantly higher among triplet and higher-order births compared to twins, which, in turn, were significantly higher than the singleton. The mean GA-expressed as mean (SD)- for singleton was significantly higher at 38.30 (2.21) weeks of GA compared to twin (36.39(4.21)) and triplets or higher-order multiples (32.72(4.14)). Thismean GA in our study was similar to another large study from the United States, which reported mean GA as 39 weeks in singleton, 35.8 weeks in twin, and 32.5 weeks in triplet births [29]. Similarly, in each BW category below 2500 g, the representation of twin, triplet, or higher-order multiple births was significantly higher than singleton (Table 1). IUGR or SGA babies were significantly higher among all multiple gestation births compared to singleton. This data underlines the previous reports suggesting that the incidence of preterm and LBW deliveries increases with multiple gestation births [17, 18, 29].

### Morbidities

#### Respiratory morbidities

Our data shows that the rate of short-term respiratory morbidity like RDS was significantly lower in all multiple gestation births, whereas pulmonary hemorrhage was significantly higher in all multiple gestation births compared to singleton. We also found a significantly higher incidence of BPD among triplet or higher-order multiple births compared to singleton and a similar trend in twin births when compared to singleton, but it was not statistically significant. Wadhawan et al. [30] looked at the short-term and long-term outcomes of more than 13,000 extremely LBW babies (birth weight 401–1000 g) born out of multiple births in participating centers of the Neonatal Research Network between 1996 and 2005. They reported a significantly higher need for surfactant therapy in twins and triplet births compared to singleton and also a higher need for mechanical ventilation in triplet compared to singleton births. They also had reported a higher incidence of BPD among twin births compared to triplet. These authors did not adjust for BW, GA or other confounding characteristics while reporting their short-term clinical outcomes, which could account for the difference in their outcomes compared to our results. Garg et al. [7] have published the perinatal characteristics and neonatal outcome data of preterm singleton, twin, and triplet births at 22–31 weeks’ gestation from Australia during the years 1994–2005 after adjusting for birth weight percentile, GA and other population-based characteristics. In this study, twins were more likely to have hyaline membrane disease compared to singletons. Surprisingly they have reported a lower rate of BPD in twins compared to the other groups. Neonatal practices and outcomes vary across the world. The neonatal practices in Australia in 1990’s and early 2000’s may be different from the United States in the years 2000–2016, which could explain the difference in outcomes. In addition, adherence to better practices and bundles, including the use of antenatal steroids, exogenous surfactant administration, early nasal continuous positive airway pressure (NCPAP), and other lung-protective strategies during mechanical ventilation, have improved the respiratory morbidities of preterm neonates leading to a difference in the outcomes reported during this study period between 2000 and 2016 [31–35].

#### Intraventricular hemorrhage and periventricular Leukomalacia

In our study, the incidence of any IVH was significantly lower in twin compared to singleton births, whereas the incidence of severe IVH (grades 3 and 4) was significantly higher in twin births compared to singleton. The difference in the incidence of any IVH or severe IVH was not statistically significant in triplet or higher-order multiple births compared to singleton. Kaufman and colleagues [36] reported an increased incidence of mild IVH in triplet birthscompared to singleton. Garg et al. [7] have reported similar severe IVH rates between singleton, twin, and triplet births, whereas Yee and group [37] reported a lower risk of IVH in triplet infants with BW < or = 1250 g, compared to singletonsand twinpatientswith similar BW and GA. The difference in outcomes between these studies could be related to the variation in the populations studied or the changes in the management practices within different neonatal units across the world over time.

The incidence of PVL was significantly lower in triplet or higher-order multiple birthscompared to singleton, whereas there was no difference in PVL incidence when comparing twin gestation births to singleton. This was in contrast to Resch et al. [38], who reported a significantly higher incidence of PVL in twin and triplet births, which may be explained due to this study reporting rates of PVL per pregnancy as opposed to per discharge record in our study. In addition, their sample size was relatively small. The protective effects of antenatal steroids in preventing IVH is well documented. Higher antenatal steroid coverage may be one of the reasons for lower IVH and PVL in triplet or higher-order multiple births. Over the last several years, changes in the neonatal practices, including better ventilation strategies [33, 39, 40] and adherence to various IVH prevention bundles [41, 42], are shown to be associated with an improvement in the incidence of IVH and PVL among preterm patients.

#### Sepsis and necrotizing enterocolitis

In our study, the incidence of bacterial sepsis was significantly lower in twin gestation births compared to singleton. While other studies have reported similar rates of sepsis in multiple gestation births compared to singleton [43–45], the large sample size and adjustment for baseline characteristics may have allowed for this study to detect differences not previously noted.

The incidence of NEC was not significantly different between any of the three groups, although the severe NEC was marginally higher in the triplet or higher-order multiple groups. Similar to our study, many of the previous studies have reported comparable rates of NEC among twins and triplets [20, 37, 44, 45]. Similar to other care practices in neonatology, during this study period, changes in neonatal feeding practices and central line care bundles may have significantly modified the neonatal outcomes such as NEC and sepsis [34, 35, 46–52].

#### Retinopathy of prematurity

In our study, we found an increased rate of all stages of ROP in triplet or higher-order gestation births compared to singleton, although we did not find any significant difference in the incidence of severe ROP between the groups. Like our findings, Kaufman and colleagues [36] also have reported increased incidence of ROP in triplet births, but they also found an increased incidence of severe ROP in triplet compared to singleton births. Some other authors [45, 53] have also reported a significantly higher rate of advanced ROP (stages II-III) in singleton compared to multiple gestation births. Consistent with our findings, Garg et al. [7] have also reported a similar incidence of severe ROP among singleton, twin, and triplet births. There has been an overall decline in the incidence of severe ROP in recent years [54, 55], which may have contributed to the inability to detect differences in severe ROP in our study. The incidence of stage 3, 4, or 5 ROP was very low in our study (< 0.40% in all groups). Improvements in neonatal practices including better oxygen saturation targeting which minimize repeat episodes of alternating hypoxia and hyperoxiamay have led to a change in the incidence of severe ROP requiring intervention [34, 35, 54, 56, 57].

### Mortality

We found a higher incidence of mortality for twin and triplet births compared to singleton among term patients, even after adjusting for baseline and hospital characteristics. In the preterm group, our analysis showed that adjusted odds of mortality were higher for triplet and higher-order births compared to singleton. However, the adjusted odds of mortality were similar for twin and singleton births. Heino et al. analyzed the neonatal death from the Euro-Peristat project, which included 5 million births from 29 countries and reported a pooled relative risk of 7.0 (95% Cl 6.1–8.0) for neonatal mortality among multiple gestation births compared to singleton [15]. Martin et al. reported that mortality was higher for multiple gestation births compared with singleton when analyzing the birth data in the US for 1980–97 [18]. Shinwell et al. also have reported an increased risk of death among triplet births on their analysis on Israel’s national very low birth weight (VLBW) infant database [58].

Several other reports suggested no significant increased risk of mortality among multiple births. In a retrospective cohort study, which was matched for GA, sex and country of birth, Shah et al. analyzed the outcomes of a total of 6079 triplets and 18,232 singletons of 24 to 32 weeks’ gestation or 500 to 1499 g [24]. This study reported no significant difference in the primary outcome between triplets and singletons. Similarly, Garite et al. [20] reported no difference in mortality between the preterm singleton, twin or triplet births at 23 to 35 weeks of gestation.

In contrast to these reports, Russell et al., using National Center for Health Statistics data of the United States from 1980 to 1999, showed that VLBW and moderately LBW multiple gestation infants had a lower mortality rate than for singletons in similar BW categories [59]. Jacquemyn et al. also have reported lower perinatal mortality in twin births compared to singleton [43]. The use of different statistical methods among studies may account for variations in results.

### Length of stay

We found that the median LOS was significantly longer in multiple gestation births compared to the singleton overall and also within each GA category. Only a very few studies with a relatively smaller sample size have compared the LOS between singleton and multiple gestation births, including twin, triplet and higher-order multiples. Vachharajani et al. [60], reported that twin and triplet births had a longer LOS compared to singleton patients born at 34 weeks, but the LOS for twin and triplet births was comparable to that of singleton at 35 and 36 weeks. A few studies did not find any difference between singleton and multiple gestation births regarding the LOS. Yee and colleagues have reported no difference in the mean LOS for twin and triplet births compared with singleton [37]. They had > 1700 babies in their analysis and included patients < 1250 g. Qiu et al. [45] and Maayan-Metzger et al. [44] also found no difference in the duration of NICU stay between the singleton and multiple gestation births.

When we analyze the data from a large population-based data set, such as KID, demographic variables like the distribution of sex, race, primary insurance or payer, and the type of hospital may be significantly different among the groups. In addition, there are wide variations in neonatal practices, including prenatal care, resuscitation guidelines, ventilation strategies, and nutritional management among the newborn nurseries across the country. These differences in socio-demographic parameters and patient care variations may have played a significant role in variable outcomes..

#### Limitations

This study has limitations due to the retrospective use of an administrative database. The findings heavily rely on the accurate reporting of the ICD codes and other variables. There are additional limitations in the case of transfers since discharge records are not linked in KID. We accounted for this limitation by excluding discharge records with indicators for “transfer in” or “transfer out.” In doing so, it is possible that the LOS reported was inadvertently shorter since the sicker patients would be more likely to undergo transfer, and the LOS from the multiple hospitalizations were not combined in the database. It is also worth noting that the gestational age ICD codes used for analysis in this dataset cover a period of 2 weeks prompting GAbreakdown in two-week categories. So the data has to be interpreted accordingly and not used for individual gestational ages. In addition, KID does not include neurodevelopmental outcomes; hence they were not analyzed. Furthermore, evidence exists to reflect the evolution of neonatal care over the years, including the years analyzed in this study between 2000 and 2016. This study could not account for advancements in clinical practice which could change the outcome of these patients over time.

## Conclusion

This study is the largest in the United States, which compared morbidity, mortality, and LOS outcomes between singleton, twin, triplet, and higher-order multiple births. Twin and triplet gestation patients are born at a younger GA compared to singleton. Multiple gestation births remain at higher risk for various morbidities and mortality compared to singletons, even after adjusting for baseline characteristics. The LOS for multiple gestation births is higher compared to singleton of the similar GA.

## Supplementary Information

**Additional file 1.**

## Data Availability

The entire data set that was used for analysis is publically available for purchase at https://www.hcup-us.ahrq.gov/kidoverview.jsp through Agency for Healthcare Research and Quality. The datasets used and/or analyzed during the current study are available from the corresponding author on reasonable request.

## Abbreviations

KID: Kids’ inpatient database
IUGR: Intra Uterine Growth Restriction
SGA: Small for Gestational Age
LBW: Low Birthweight
LOS: Length of Stay
PMA: Post Menstrual Age
RDS: Respiratory Distress Syndrome
NEC: Necrotizing Entero Colitis
IVH: Intra Vnetricular Hemorrhage
PVL: Periventricular leukomalacia
ROP: Retinopathy of prematurity
BPD: Bronchopulmonary dysplasia
GA: Gestational Age
BW: Birth Weight
