# Supplementary material for: Outcomes of multiple gestation births compared to singleton: analysis of multicenter KID database
Source: Matern Health Neonatol Perinatol. 2021 Oct 28;7:15. doi: 10.1186/s40748-021-00135-5 (PMC8554969; doi:10.1186/s40748-021-00135-5)
Supplement: Supplementary file 1 — Additional file 1. [file 40748_2021_135_MOESM1_ESM.docx]

**Supplementary information:**

Appendix 1:

1. ICD code list:

| **Code category** | **ICD-9 CM Diagnosis code** | **ICD-10 CM Diagnosis code** | **Clinical Classification software diagnosis category** |
| --- | --- | --- | --- |
| Bacterial sepsis | 7718* | P36* |  |
| Biliary atresia | 75161 | Q442-Q443 |  |
| Birth weight | V2131, 76501, 76511, V2132, 76502, 76503, 76512, 76513, V2133, 76504, 76505, 76514, 76515, V2134, 76506, 76507, 76516, 76517, V2135, 76508, 76518, 76509, 76519, V2130, 76500, 76510 | P0701, P0501, P0511, P0702, P0703, P0502, P0503, P0512, P0513, P0714, P0715, P0504, P0505, P0514, P0515, P0716, P0717, P0506, P0507, P0516, P0517, P0718, P0508, P0518, P0509, P0519, P0700, P0710, P0500, P0510 |  |
| Bronchopulmonary dysplasia | 7707 | P270, P271, P278 |  |
| Comfort care | V667 | Z515 |  |
| Congenital anomalies of urinary system | 753* | Q60*, Q61*, Q62*, Q63*, Q64* |  |
| Congenital diaphragmatic hernia | 7566 | Q790 |  |
| Congenital neuro anomalies |  |  | 216 |
| Critical congenital heart disease | 74602, 7463, 74710, 74711, 7453, 74511, 7462, 7452, 74601, 74731, 7461, 7450, 74510, 7467, 74741 | Q221, Q230, Q251, Q2521, Q204, Q201, Q225, Q213, Q220, Q255, Q229, Q200, Q203, Q234, Q262 |  |
| Delivery type | 650, V3000, V301, V302, V3100, V311, V312, V3200, V321, V322, V3300, V331, V332, V3400, V341, V342, V3500, V351, V352, V3600, V361, V362, V3700, V371, V372, V3900, V391, V392, V3001, V3101, V3201, V3301, V3401, V3501, V3601, V3701, V3901 | O80, Z3800, Z3830, Z3861, Z3863, Z3865, Z3868, Z381, Z384, Z387, O82, Z3801, Z3831, Z3862, Z3864, Z3866, Z3869 |  |
| Downs syndrome | 7580 | Q90* |  |
| Gastroschisis omphalocele prune belly | 75671-75673 | Q792-Q794 |  |
| Gestational age | 76521, 76522, 76523, 76524, 76525, 76526, 76527, 76528, 76529, 76520 | P0721, P0722, P0723, P0724, P0725, P0726, P0731, P0732, P0733, P0734, P0735, P0736, P0737, P0738, P0739, P0720, P0730 |  |
| Hirschsprung disease | 7513 | Q431 |  |
| IUGR or SGA | 764* | P05* |  |
| Intraventricular haemorrhage | 77211, 77212, 77213, 77214, 77210 | P520, P521, P5221, P5222 |  |
| Multiples | V3000, V3001, V301, V302, V3100, V3101, V311, V312, V3200, V3201, V321, V322, V3300, V3301, V331, V332, V3400, V3401, V341, V342, V3500, V3501, V351, V352, V3600, V3601, V361, V362, V3700, V3701, V371, V372, V3900, V3901, V391, V392 | Z3800, Z381, Z3801, Z3830, Z384, Z3831, Z3861, Z3863, Z3865, Z3868, Z387, Z3862, Z3864, Z3866, Z3869, Z3800, Z381, Z3801, Z3830, Z384, Z3831, Z3861, Z3862, Z3863, Z3864, Z3865, Z3866, Z3868, Z387, Z3869 |  |
| Necrotizing enterocolitis | 77751, 77752, 77753, 7776, 77750 | P771, K5531, P772, K5532, P773, K5533, P780, P779, K5530 |  |
| Osteogenesis imperfecta | 75651 | Q780 |  |
| Periventricular leukomalacia | 7797 | P912 |  |
| Pulmonary hemorrhage | 7703 | P26* |  |
| Respiratory distress syndrome | 769 | P220 |  |
| Retinopathy of prematurity | 36220, 36223, 36224, 36225, 36226, 36227, 36221, 36220, 36222, 36223, 36224, 36225, 36226, 36227, 36221 | H3510*, H3512*, H3513*, H3514*, H3515*, H3516*, H3517*, H3510*, H3511*, H3512*, H3513*, H3514*, H3515*, H3516*, H3517* |  |
| Spina bifida | 741* | Q05* |  |
| Tracheoesophageal fistula | 7503 | Q390-Q394 |  |
| Trisomy 13 18 | 7581-7582 | Q91* |  |

1. List of congenital anomalies excluded:

They were excluded if they had ICD codes for chromosomal disorders such as trisomy 13, 18, or 21, or congenital anomalies of the central nervous system (including meningocele, meningomyelocele, spina bifida, anencephaly, and other congenital nervous system malformations). They were also excluded if they had critical congenital heart diseases (which did not include minor defects such as patent ductus arteriosus, atrial septal defects, and ventricular septal defect), osteogenesis imperfecta, and other anomalies like tracheoesophageal fistula, diaphragmatic hernia, gastroschisis, omphalocele, prune belly syndrome, Hirschsprung’s disease, biliary atresia, and congenital malformations of the urinary system as these congenital anomalies itself can lead to higher mortality. Some minor congenital anomalies may not change the LOS, while other congenital anomalies such as gastroschisis could have a wide range of impact on LOS.([48](#_ENREF_48)) We excluded congenital anomalies to remove such confounding in results.

Appendix 2:

| <24 weeks | 23 |
| --- | --- |
| 24 weeks | 24.5 |
| 25-26 weeks | 26 |
| 27-28 weeks | 28 |
| 29-30 weeks | 30 |
| 31-32 weeks | 32 |
| 33-34 weeks | 34 |
| 35-36 weeks | 36 |
| >37 weeks | 38.5 |
